# Structural insights into disease-associated mutations in the microRNA processing machinery

**DOI:** 10.1038/s12276-026-01669-4

**Published:** 2026-03-06

**Authors:** Hansol Lee, Jaehyun Lee, Soung-Hun Roh

**Affiliations:** 1 School of Biological Sciences, Seoul National University, Seoul, Republic of Korea; 2 Institute of Molecular Biology and Genetics, Seoul National University, Seoul, Republic of Korea; 3 Department of Biotechnology and Bioinformatics, College of Science and Technology, Korea University, Sejong, Republic of Korea; 4 Interdisciplinary Major Program in Bio Medical and Data Science Convergence, Korea University, Sejong, Republic of Korea

**Keywords:** RNAi, Oncogenes, Protein folding

## Abstract

MicroRNAs (miRNAs) are small noncoding RNAs that mediate post-transcriptional gene silencing through a conserved pathway involving the sequential actions of DROSHA, DICER and Argonaute proteins. These RNA interference core components recognize and process precursor transcripts with structural precision to generate functional miRNA duplexes and guide-loaded Argonaute effector complexes. Recent genetic and structural studies have revealed disease-associated mutations in these proteins, particularly within their catalytic centers and RNA-binding interfaces, that impair miRNA biogenesis and contribute to human pathologies. Such mutations disrupt RNA cleavage fidelity, destabilize domain architecture or hinder small RNA loading, leading to cancers and developmental disorders, including Wilms tumor, DICER1 syndrome, myelodysplastic syndromes and Lessel–Kreienkamp syndrome. This Review highlights the structural basis of these pathogenic mutations and discusses how emerging insights from structural biology are shaping our understanding of RNA interference-related disease mechanisms and guiding potential therapeutic strategies.

## Understanding RNAi disorders through structural biology advances

MicroRNAs are small molecules that help control how genes work in our bodies. They are made through a complex process involving proteins such as DROSHA, DICER and AGO2. These proteins work together to ensure microRNAs are made correctly. However, changes or mutations in these proteins can lead to diseases such as cancer and developmental disorders. Researchers studied how these mutations affect the proteins’ structure and function. They used advanced techniques to see how mutations change the proteins’ shape and ability to work properly. For example, mutations in DROSHA can stop it from cutting RNA correctly, leading to problems in gene regulation. Similarly, changes in DICER can affect its ability to process RNA, which is crucial for preventing tumors. The study shows that understanding these mutations helps us learn how they cause diseases.

This summary was initially drafted using artificial intelligence, then revised and fact-checked by the author.

## Introduction

MicroRNAs (miRNAs) are ~22-nucleotide noncoding RNAs that regulate gene expression post-transcriptionally by guiding Argonaute (AGO) proteins within the RNA-induced silencing complex (RISC) to target mRNAs involved in diverse biological processes, including development, differentiation, immune responses and oncogenesis^1,2^.

The biogenesis of miRNAs is a tightly regulated process involving sequential cleavage of primary and precursor miRNA transcripts by the RNase III enzymes DROSHA and DICER, respectively, followed by loading of the mature miRNA duplex into AGO proteins^3–12^. The catalytic and RNA-binding activities of these proteins rely on intricate domain architectures, including dual RNase III domains in DROSHA and DICER and PAZ, MID and PIWI domains in AGO. Biochemical and structural studies underscore the important role of conformational plasticity and allosteric regulation in maintaining pathway fidelity^13–17^. Advances in high-resolution cryo-electron microscopy (cryo-EM) and molecular dynamics simulations have further elucidated the impact of disease-associated mutations on the structural and functional integrity of the RNA interference (RNAi) machinery. These approaches have revealed dynamic domain rearrangements and mutation-sensitive interfaces underlying miRNA biogenesis and RISC activation, offering mechanistic insights with therapeutic implications.

Genetic studies have increasingly implicated germline and somatic mutations in DROSHA, DICER1 and AGO2 in a broad spectrum of human diseases, including Wilms tumor, endometrial and thyroid cancers, DICER1 tumor predisposition syndrome (DTPS), myelodysplastic syndromes (MDS) and neurodevelopmental disorders such as Lessel–Kreienkamp syndrome^18–23^. Notably, many pathogenic variants cluster within evolutionarily conserved structural cores essential for enzymatic activity and RNA binding, such as the RIIIDb active site of DICER, the RIIIDa/b interface of DROSHA and the PAZ, MID and PIWI domains of AGO2^24–28^.

In this Review, we integrate emerging structural and genetic insights to address how disease-associated mutations disrupt the molecular architecture and enzymatic logic of the miRNA processing machinery. We systematically examine pathogenic mutations in DROSHA, DICER and AGO2 and discuss how these mechanistic insights provide a framework for structure-guided therapeutic strategies aimed at restoring or modulating RNAi pathway activity in disease contexts.

## Overview of the structural features of DROSHA, DICER and AGO proteins

**Fig. 1:**
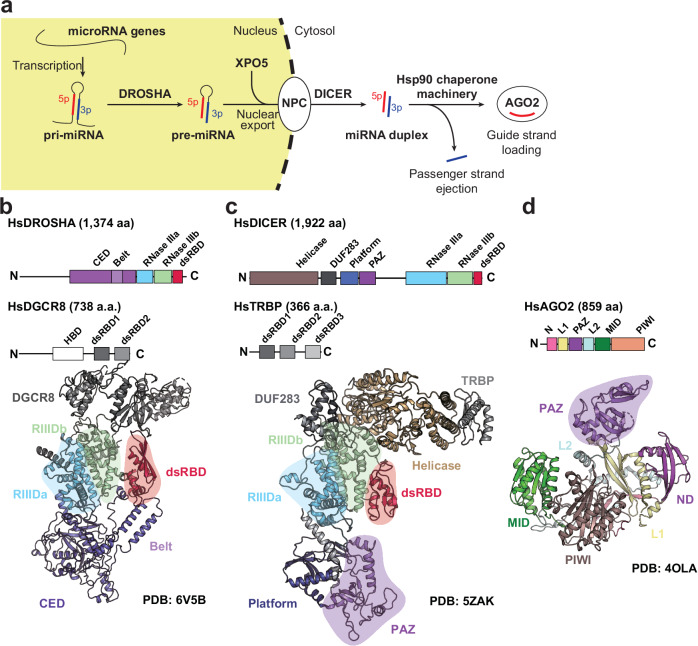
Functional roles and domain organization of human RNAi machinery. **a** Schematic overview of the RNAi pathway in human cells. pri-miRNA transcripts are processed in the nucleus by the DROSHA–DGCR8 complex into precursor miRNAs (pre-miRNAs), which are then exported to the cytoplasm by Exportin-5 (XPO5). In the cytoplasm, DICER, assisted by the cofactor TRBP, cleaves pre-miRNAs into ~22-nt miRNA duplexes. The guide strand is subsequently loaded into AGO2 via the Hsp90 chaperone machinery, while the passenger strand is ejected, completing the formation of the RISC. **b** Domain architecture and three-dimensional (3D) structure of human DROSHA and its cofactor DGCR8. Drosha contains an arginine/serine (R/S)-rich region, a central domain (CED), a Belt motif, RIIIDa/b and a C-terminal dsRBD. DGCR8 comprises a heme-binding domain (HBD) and two dsRNA-binding domains (dsRBD1 and dsRBD2). The structure shown is the human DROSHA–DGCR8–pri-miRNA complex (PDB: 6V5B). Major domains are highlighted and colored according to the schematic above. **c** Domain architecture and 3D structure of human DICER and its cofactor TRBP. Dicer contains an N-terminal helicase domain, a DUF283 domain, a platform domain, a PAZ domain, tandem RIIIDa/b domains and a C-terminal dsRBD. TRBP contains three dsRBDs, with dsRBD3 mediating protein–protein interaction. The structure is the human DICER–TRBP complex bound to pre-miRNA (PDB: 5ZAK). Functional domains are labeled and colored consistently. **d** Domain architecture and structure of human AGO2 in complex with a miRNA guide strand (PDB: 4OLA). AGO2 consists of four structured domains—N, PAZ, MID and PIWI—connected by two linker regions (L1 and L2). The guide strand is anchored at both ends by the PAZ and MID domains, while the PIWI domain harbors the RNase H-like catalytic center responsible for target RNA slicing.

The core components of the miRNA pathway, DROSHA, DICER and Argonaute2 (AGO2), are multidomain ribonucleases and RNA-binding proteins that function sequentially and in a highly coordinated manner to mediate miRNA biogenesis and gene silencing^13–22^ (Fig. 1a). Each protein plays a distinct role at specific stages of the pathway, collectively ensuring the maturation, loading and functional deployment of miRNAs for post-transcriptional gene regulation. Despite their distinct functions, these proteins share conserved RNA-recognition motifs and use structurally integrated mechanisms for RNA cleavage and guide strand handling.

DROSHA operates in the nucleus and initiates miRNA biogenesis by cleaving long primary miRNA (pri-miRNA) transcripts into pre-miRNAs^29^. This step is catalyzed by the microprocessor complex, which is composed of DROSHA and its essential cofactor, DGCR8. Structurally, DROSHA consists of an N-terminal RS-rich region, a conserved central domain (CED), a double-stranded RNA (dsRNA)-binding domain (dsRBD) and a C-terminal tandem RNase III domain pair, designated RIIIDa and RIIIDb^13,29^ (Fig. 1b). These RNase III domains form an intramolecular pseudo-dimer that cleaves both strands of the pri-miRNA stem–loop, generating a 3′ overhang of two nucleotides required for subsequent recognition by DICER^29,30^. DGCR8 contains an N-terminal heme-binding domain and two C-terminal dsRNA-binding domains (dsRBD1 and dsRBD2), which recognize the basal junction of the pri-miRNA and stabilize the Microprocessor complex. The interaction between DROSHA’s RNase III domains and DGCR8’s dsRBDs ensures precise cleavage-site positioning, with DGCR8 anchoring and aligning the pri-miRNA within DROSHA’s catalytic groove (Fig. 1b).

DICER, a cytoplasmic RNase III enzyme, catalyzes the second step in miRNA maturation by processing pre-miRNAs into ~22-nucleotide miRNA duplexes^17^. Compared with DROSHA, DICER exhibits greater structural complexity, comprising an N-terminal helicase domain involved in substrate discrimination and unwinding, a DUF283 domain, a PAZ domain that binds the 3′ overhang of pre-miRNAs, a tandem RIIIDa/b catalytic module and a C-terminal dsRBD^10^ (Fig. 1c). The spatial relationship between the PAZ and RNase III domains functions as a molecular ruler, enabling DICER to cleave RNA substrates at a defined distance from the PAZ-binding site to ensure uniform length^5,10^. TRBP (TAR RNA-binding protein), a key cofactor, contains three dsRBDs: dsRBD1 and dsRBD2 mediate RNA binding, while dsRBD3 facilitates protein–protein interaction. TRBP interacts with DICER’s helicase and DUF283 domains to stabilize the pre-miRNA substrate and enhance processing efficiency by tethering the substrate near the active site (Fig. 1c).

AGO2 is the principal catalytic component of the RISC. It binds the guide strand of mature miRNAs and mediates gene silencing either through endonucleolytic cleavage (slicing) or translational repression of target messenger RNAs^31^. AGO2 consists of four conserved domains: an N-terminal domain involved in duplex unwinding and structural regulation, a PAZ domain that anchors the 3′ end of the miRNA guide strand, a MID domain that secures the 5′ phosphate of the guide strand, and a PIWI domain that adopts an RNase H-like fold and harbors the catalytic residues required for slicing mRNA cleavage^32,33^ (Fig. 1d).

Despite their distinct roles, DROSHA, DICER and AGO2 share conserved architectural features that enable the precise recognition of RNA substrates^17^. DROSHA and DICER both use tandem RNase III domains (RIIIDa and RIIIDb) to cleave dsRNA. DICER uniquely contains a PAZ domain and helicase module, which facilitate precise length control during substrate processing. The PAZ domain, a common structural element in DICER and AGO2, adopts an Oligonucleotide Binding (OB)-fold that anchors RNA termini. In DICER, it binds the 3′ end of pre-miRNAs, whereas in AGO2, it secures the 3′ end of mature guide RNAs^32^ (Fig. 1b–d).

Catalytic activity in DROSHA and DICER relies on divalent metal ion coordination within RNase III active sites for phosphodiester bond cleavage^3^. AGO2 mediates target mRNA cleavage through its RNase H-like PIWI domain, which requires extensive guide–target base pairing and precise spatial alignment of the RNA duplex within the catalytic groove^32^. All three proteins bind RNA via positively charged grooves and structured loops in a largely sequence-independent manner. AGO2 uniquely distinguishes between guide and passenger strands through conformational checkpoints during RISC assembly^34^.

These structural features provide a mechanistic framework for understanding the impact of mutations in conserved or divergent domains on RNA recognition, catalytic fidelity and downstream gene regulation.

## Structural basis of disease-associated mutations in DROSHA

### Domain organization and catalytic mechanism

**Fig. 2:**
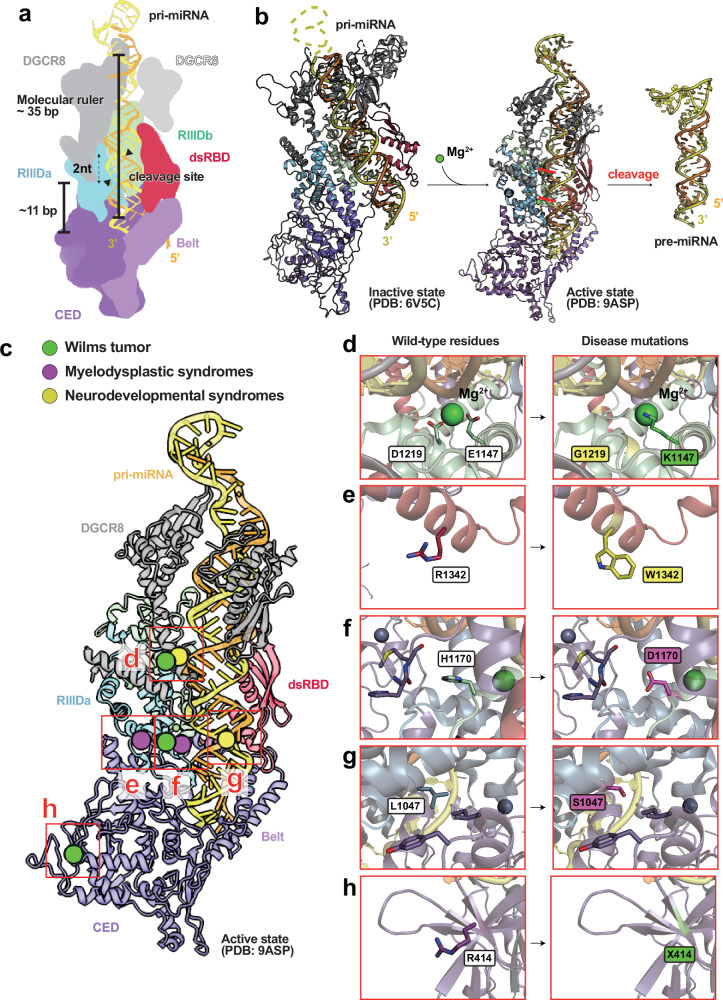
Functional mechanism and disease-associated mutation hotspots of DROSHA. **a** Schematic representation of the domain organization of human DROSHA bound to pri-miRNA. The molecular model is based on the DROSHA–DGCR8–pri-let-7a complex (PDB: 9ASP). Protein coloring follows the scheme used in Fig. 1b. The 5p strand of the duplex region of pri-miRNA is shown in orange, and the 3p strand including the terminal loop is shown in yellow. The RNase III cleavage sites are indicated by black wedges. The length of the stem duplex and the number of base pairs from the basal junction to the cleavage sites are also annotated. **b** Comparison of inactive and active structural states of DROSHA. The inactive-state model was derived from PDB: 6V5C, and the active-state model from PDB: 9ASP. Domain coloring is consistent with **a**. **c** Structural mapping of disease-associated mutations in DROSHA. The DROSHA–DGCR8–pri-let-7a complex (PDB: 9ASP) is shown with mutation sites associated with Wilms tumor, MDS and neurodevelopmental syndromes highlighted in green, magenta and yellow, respectively. **d**–**h** Close-up views of representative pathogenic mutations. Each panel shows the wild-type residue (left) and the corresponding mutated residue (right). Domain coloring is consistent with **a**. The D1219G and E1147K mutations (**d**), R1342W (**e**), H1170D (**f**), L1047S (**g**) and R414X truncation (**h**) are depicted. X denotes a stop codon. A coordinated magnesium ion (Mg^2+^) is shown as a green sphere.

Human DROSHA comprises an N-terminal proline-rich region, a CED, a dsRBD and two C-terminal RNase III domains (RIIIDa and RIIIDb) that form a catalytic pseudo-dimer (Fig. 2a). Recent cryo-EM studies revealed that a two-stranded coiled coil within the CED, termed the Belt, binds the basal junction of pri-miRNA by forming a four-way junction with the dsRBD, C-terminal peptide and the Wedge. This configuration rigidly couples the dsRBD and RNase III domains, stabilizing the interaction with the pri-miRNA to ensure efficient and precise cleavage. The dsRBD serves as a molecular caliper, measuring the distance along the RNA stem between the basal and apical junctions. The positioning of these dsRBDs near the apical junction appears to be independent of DROSHA’s catalytic core, as demonstrated in an alternate, partially docked structural conformation^13,14,35^ (Fig. 2a).

The pri-miRNA initially adopts an inactive conformation. Upon coordination of magnesium ions at the RNase III catalytic sites, the complex undergoes structural activation, enabling precise cleavage of the RNA stem–loop (Fig. 2b). The cleavage site is primarily determined by the structural configuration of the pri-miRNA rather than by specific sequence motifs. DGCR8 recognizes the double-stranded stem and flanking single-stranded regions of the pri-miRNA, positioning DROSHA approximately 11 base pairs from the junction between the single-stranded basal region and the stem–loop^36^. At this site, the RNase III domains coordinate two magnesium ions through four conserved acidic residues (E1147, D1151, D1369 and E1373) to catalyze pri-miRNA cleavage, generating ~2-nucleotide 3′ overhangs^13,14,35^. Accurate alignment of the pri-miRNA within the catalytic valley formed by RIIIDa and RIIIDb is essential for processing fidelity. Each RNase III domain contributes one catalytic site, forming a functional pseudo-dimer.

### Mutation hotspots and disease associations on DROSHA structure

Pathogenic mutations in human DROSHA are increasingly recognized as drivers of tumorigenesis and developmental disorders. Structural analyses reveal that these mutations frequently localize to the RNase III catalytic cores, RNA-binding grooves and interdomain interfaces, creating specific mechanical failures in pri-miRNA processing.

The E1147K mutation in the RIIIDb is identified in approximately 12% of Wilms tumor cases^18,37^. Structurally, E1147 is a conserved acidic residue essential for coordinating Mg²⁺ ions within the catalytic valley. The substitution with lysine (K) reverses the charge, abolishing the endonucleolytic activity of the RIIIDb domain^38,39^. Because the Microprocessor functions as a heterotrimer (one DROSHA and two DGCR8 molecules), these catalytically inactive DROSHA mutants can still bind DGCR8 and pri-miRNA substrates. These mutations exert dominant-negative effects by disrupting the intramolecular RIIIDa/b pseudo-dimer that is essential for coordinated pri-miRNA cleavage. Mutations such as E1147K in the RIIIDb destabilize this catalytic unit, rendering the Microprocessor complex inactive even in the presence of wild-type DROSHA. Because each DGCR8-associated Microprocessor contains a single DROSHA molecule, incorporation of a catalytically defective protein leads to global impairment of pri-miRNA processing and widespread miRNA depletion.

Other sporadic Wilms tumor mutations, such as R414X (where X denotes a stop codon resulting in truncation) lead to the loss of the C-terminal catalytic domains entirely^39^. While these truncated proteins cannot cleave RNA, they may disrupt the stoichiometry of the Microprocessor complex. Conversely, missense mutations such as R1342W, identified in neurodevelopmental syndromes, are located on the protein surface^40^. While less intuitively damaging than active-site mutations, R1342W probably disrupts regulatory protein–protein interactions or secondary structure formation essential for Microprocessor stability^40,41^.

DROSHA mutations are associated with hematopoietic disorders, notably MDS, a group of clonal bone marrow disorders characterized by ineffective hematopoiesis, peripheral cytopenias and progression to acute myeloid leukemia^42^. Missense mutations such as L1047S and H1170D impair DROSHA-mediated miRNA maturation, altering the expression of hematopoietic lineage regulators and contributing to dysplastic and stem cell exhaustion.

In the nervous system, de novo heterozygous mutations in DROSHA, including D1219G and R1342W, have been identified in patients with neurodevelopmental syndromes, characterized by intellectual disability, hypotonia and speech delay^40^. These mutations impair the processing of neuronal miRNAs essential for cortical development, synaptogenesis and neural circuit formation. Disruption of canonical pathways alters brain gene expression, leading to neurodevelopmental impairment and reduced neural connectivity.

Structural and computational analyses have demonstrated that disease-associated DROSHA mutations frequently localize to functionally essential regions, including the RNase III catalytic cores, RNA-binding grooves and interdomain interfaces (Fig. 2c). These regions are crucial for substrate recognition and catalytic precision. The E1147K mutation in Wilms tumor and the D1219G mutation in neurodevelopmental syndromes each substitute a catalytic glutamate or aspartate residue within the RIIIDb domain, which is necessary for magnesium ion coordination. These substitutions abolish endonucleolytic activity, resulting in widespread miRNA depletion^37–39^ (Fig. 2d). In MDS, the L1047S and H1170D mutations alter local electrostatic properties. H1170D may disrupt hydrogen bonding near the catalytic site, while L1047S introduces a polar side chain into a hydrophobic region, destabilizing the local structure (Fig. 2f, g). Certain mutations present challenges for structural interpretation. The R414X mutation in Wilms tumor and the R1342W mutation in neurodevelopmental syndromes are located on the surface of the protein (Fig. 2e, h), suggesting potential effects on secondary structure formation or regulatory protein–protein interactions.

Molecular dynamics simulations have shown that mutations such as I625K, L1047S and H1170D reduce DROSHA’s structural stability by increasing system energy and impairing RNA substrate binding^41^. I625K introduces a salt bridge within a flexible loop, potentially misaligning the RNA-binding interface. L1047S disrupts α-helical hydrogen bonding, weakening domain packing. H1170D destabilizes the RNase IIIb fold by interfering with aromatic π–π stacking near the catalytic groove.

Collectively, these mutations impair pri-miRNA processing and contribute to oncogenic, hematologic and neurodevelopmental disorders by disrupting post-transcriptional gene silencing. These findings emphasize the critical role of structural integrity in DROSHA’s catalytic function and identify key hotspots for disease-associated mutations.

## Structural basis of disease-associated mutations in DICER

### Domain architecture and catalytic mechanism

**Fig. 3:**
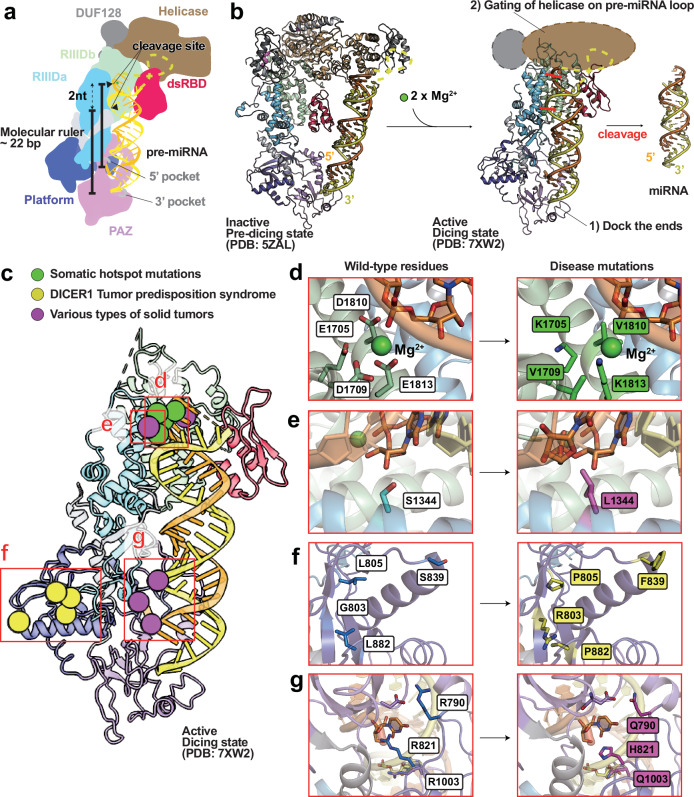
Functional mechanism and disease-associated mutation hotspots of DICER. **a** The molecular model is based on the DICER–pre-let-7a complex (PDB: 7XW2). Protein coloring follows the scheme used in Fig. 1b. The 5p strand of the miRNA duplex is colored in orange, and the 3p strand in yellow. The terminal loop, which is unresolved in the structure, is represented as a dashed yellow line. RNase III cleavage sites are indicated by black wedges. The length of the resulting miRNA duplex (~22 nucleotides) and the characteristic 3′ two-nucleotide overhang are annotated, reflecting DICER’s role as a molecular ruler that defines cleavage distance based on the spacing between the PAZ and RNase III domains. **b** Comparison of inactive and active conformational states of DICER. The inactive-state model is derived from PDB: 5ZAL, and the active-state model from PDB: 7XW2. Domain coloring is consistent with **a**. **c** Structural mapping of disease-associated mutations in DICER. The active DICER structure (PDB: 7XW2) is shown with mutation sites associated with somatic hotspot mutations, DTPS and various solid tumors colored in green, yellow, and magenta, respectively. **d**–**g** Close-up views of representative pathogenic mutations. Each panel shows the wild-type residue (left) and the corresponding mutant residue (right). Coloring of wild-type residues follows the domain scheme in **a**, while mutant residues are colored according to the disease categories defined in **c**. The following mutations are depicted: D1709V, E1705K, D1810V, and E1813K (**d**); S1344L (**e**); G803R, L805P, S839F and L882P (**f**); R790Q, R821H and R1003Q (**g**). A coordinated magnesium ion shown as a green sphere (substituted for calcium in the model).

DICER is a multidomain RNase III endonuclease composed of an N-terminal helicase domain (Hel1/Hel2), a DUF283 domain, a PAZ domain, two RNase III domains (RIIIDa and RIIIDb) and a C-terminal dsRBD (Fig. 3a). Following the processing of pri-miRNA into pre-miRNA by DROSHA, DICER cleaves pre-miRNAs into ~22-nucleotide miRNA duplexes through the coordinated action of its RIIIDa and RIIIDb domains, which together form a composite catalytic center^10,16^. The PAZ domain anchors the 3′ two-nucleotide overhang of the pre-miRNA, while the platform and MID domains engage the 5′ phosphate, enabling precise substrate positioning. This spatial configuration establishes the ‘molecular ruler’ mechanism, whereby the cleavage sites are defined as ~22 nucleotides from the PAZ binding pocket^10^ (Fig. 3a). Each RNase III domain contributes to strand-specific cleavage: RIIIDa cleaves the 3′ arm, and RIIIDb cleaves the 5′ arm. Catalysis requires the coordination of two magnesium ions by conserved acidic residues, including E1705, D1709, D1810 and E1813 (RIIIDb) and D1320 and E1323 (RIIIDa), which mediate phosphodiester bond hydrolysis^16^. The helicase domain functions as a gating module, regulating substrate selection based on structure and length. It permits canonical pre-miRNAs (~60–70-bp hairpins) to bypass ATP-dependent remodeling while restricting access to longer or perfectly paired dsRNAs unless ATP hydrolysis occurs^16,43^. This gating mechanism ensures substrate specificity and prevents the unintended cleavage of endogenous long dsRNAs or viral RNAs (Fig. 3b).

### Diseases caused by mutations in human DICER

Mutations in the DICER1 gene cause a hereditary tumor predisposition syndrome associated with a range of benign and malignant neoplasms resulting from defective miRNA processing. Germline and somatic mutations in DICER1 define this condition, known as DICER1 syndrome^44,45^. The syndrome typically presents in childhood or adolescence and affects multiple organs, including the lungs, kidneys, ovaries, thyroid gland and central nervous system. DICER1 encodes the RNase III endonuclease essential for miRNA and small interfering RNA maturation. Most affected individuals carry a germline loss-of-function mutation in one DICER1 allele, followed by a second somatic missense mutation at catalytic residues within the RIIIDb domain (for example, E1705, D1709, G1809, D1810 and E1813) in tumor tissues. This two-hit model aligns with classical tumor suppressor gene inactivation^43^ (Fig. 3c). Somatic mutations in sporadic tumors often localize to a recurrent hotspot in the RIIIDb domain, where they compromise enzymatic activity^46^.

The most characteristic tumor in DICER1 syndrome is pleuropulmonary blastoma (PPB), a rare pediatric lung cancer that progresses from cystic to solid forms. DICER1 mutations are present in nearly all familial PPB cases^47^. Additional DICER1-associated tumors include Wilms tumor, wherein RIIIDb mutations impair miRNA processing and promote oncogenesis through deregulation of the let-7 family^38^. Sertoli–Leydig cell tumors of the ovary, which can lead to virilization in adolescent females, are also linked to DICER1 mutations^48^. Furthermore, multinodular goiter, sometimes progressing to thyroid carcinoma, is commonly observed in mutation carriers, particularly during adolescence^44^.

### Oncogenic mutations in the DICER domains

Several DICER mutations, including somatic hotspot variants and those associated with DTPS and various solid tumors, can be mapped onto the structural model of DICER (Fig. 3c). Somatic hotspot mutations in the RIIIDb domain, particularly at residues E1705K, D1709V, D1810V and E1813K, have been identified across multiple tumor types, including endometrial carcinoma and Sertoli–Leydig cell tumors^20,24,46,49^. These mutations impair the coordination of catalytic magnesium ions within the RIIIDb active site, resulting in the selective loss of 5p miRNA strand processing while preserving 3p strand processing. This strand bias contributes to the activation of the oncogenic pathway^3,49^ (Fig. 3d). In uterine corpus endometrial carcinoma and embryonal rhabdomyosarcoma-like tumors, the G1809R mutation, located adjacent to the RIIIDb catalytic center, alters the local α-helical structure, affecting RNA binding and cleavage fidelity^49^. In addition, the S1344L mutation within the RIIIDa domain, positioned at the interdomain interface with RIIIDb, is proposed to disrupt allosteric communication. This mutation has been detected in tumors with mixed histologies, such as embryonal rhabdomyosarcoma-like neoplasms^50^ (Fig. 3e).

Mutations in the platform domain, including G803R, L805P, S839F and L881P, are associated with DTPS. These mutations impair the anchoring of the 5′ phosphate of pre-miRNAs, reducing substrate alignment and processing efficiency of tumor-suppressive miRNAs^51^ (Fig. 3f). Recurrent mutations in RNA-interacting residues within the 5′ pocket of DICER have also been identified in various cancers, as reported in The Cancer Genome Atlas, including R790Q in colon adenocarcinoma, R821H in colorectal adenocarcinoma and R1003Q in uterine endometrioid carcinoma and rectal adenocarcinoma^52,53^ (Fig. 3g).

In vitro studies indicate that R821H and R1003Q mutants exhibit cleavage-site selection defects, producing heterogeneous products (21–23 nucleotides) from dsRNA substrates with canonical 3′ 2-nucleotide overhang^16^. These substitutions introduce steric clashes or disrupt backbone flexibility at the 5′ phosphate-binding interface, impairing substrate alignment. They may also destabilize domain–domain interactions or alter the electrostatic surface required for efficient RNA engagement. These structural perturbations reduce cleavage fidelity and processing efficiency, preferentially affecting the maturation of tumor-suppressive miRNAs.

These oncogenic mutations disrupt the precise coordination geometry required for symmetric cleavage by the RNase III domains, leading to strand-specific processing defects. Loss of structural stability at domain interfaces or RNA-binding surfaces compromises DICER’s ability to accurately measure and align its substrates, particularly concerning 5′ phosphate recognition. Collectively, these alterations impair the biogenesis of tumor-suppressive miRNAs and promote the selective expression of oncogenic transcripts, contributing to tumor progression.

## Structural basis of disease-associated mutations in AGO

### Domain organization and function of AGO

**Fig. 4:**
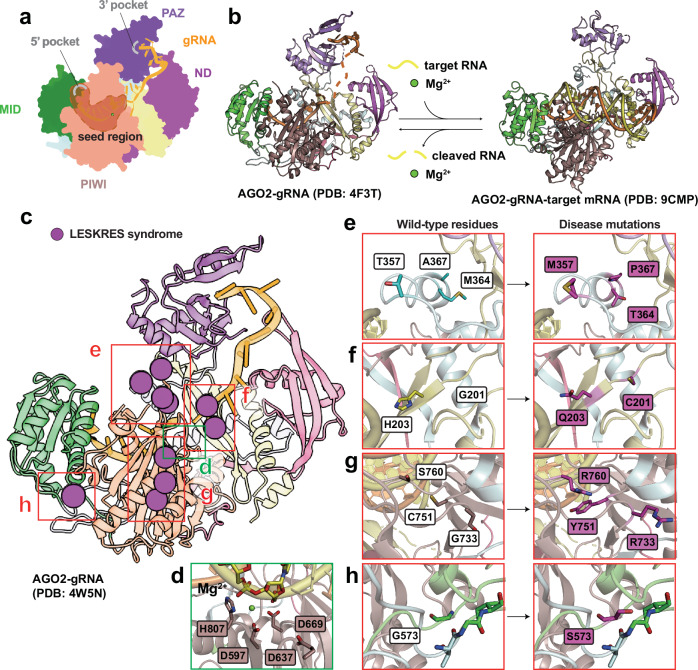
Functional mechanism and disease-associated mutation hotspots of AGO2. **a** Schematic representation of the domain architecture of human AGO2 bound to a guide miRNA. The molecular model is based on the AGO2–let-7a complex (PDB: 4F3T). Protein domains are colored according to the scheme in Fig. 1b. The single-stranded miRNA guide is shown in orange. The 5′-end binding pocket within the MID domain and the 3′-end binding pocket within the PAZ domain are depicted in gray. The seed region, positioned within the PIWI domain, is indicated by a darker-shaded region enclosed by a dashed outline. **b** Comparison of the unloaded RISC (AGO2–guide RNA complex) and the target-bound RISC complex. The RISC model is derived from PDB: 4F3T, and the target-bound RISC model from PDB: 9CMP. Domain coloring follows that o**f a**. The target RNA is shown in yellow. **c** Close-up view of the target cleavage site within the PIWI catalytic center. The cleavage site of the target RNA and the catalytic residues of AGO2—D597, E637, D669 and H807—are depicted as sticks. **d** Structural mapping of disease-associated mutations in AGO2. The target-bound RISC structure (PDB: 9CMP) is shown with mutation sites associated with LESKRES highlighted as magenta-colored spheres. **e**–**h** Close-up views of representative pathogenic mutations. Each panel displays the wild-type residue (left) and the corresponding mutant residue (right). Coloring of wild-type residues follows the domain scheme in **a**, while mutant residues are colored according to the disease category shown in **d**. The following mutations are depicted: T357M, M364T and A367P (**e**); H203Q and G201C (**f**); G733R, C751Y and S760R (**g**); G573S (**h**).

Among the four human AGO homologs (AGO1–4), AGO2 is the only catalytically active member in mammals and mediates gene silencing through a coordinated ‘slicing’ mechanism supported by its modular domain architecture^32,54^. AGO2 comprises four principal domains—N-terminal (N), PAZ, MID and PIWI, which are connected by two structured linkers (L1 and L2), forming a bilobal architecture that facilitates RNA binding, positioning and catalytic cleavage of target RNAs^55^ (Fig. 4a). Within the RISC, AGO2 binds a small RNA duplex consisting of a guide strand and a passenger strand. During loading, the passenger strand is displaced and degraded, while the guide strand is retained and anchored within AGO2’s functional domains for target recognition. The seed region of the guide RNA, typically nucleotides 2–8 from the 5′ end, is critical for initiating base pairing with complementary target sequences and largely determines target specificity. The PAZ domain anchors the 3′ end of the guide RNA, stabilizing its conformation and maintaining its alignment for target pairing^32,55^. Simultaneously, the MID domain binds the 5′ monophosphate of the guide strand, securing it for accurate target recognition^55,56^. The PIWI domain, central to AGO2’s catalytic function, adopts an RNase H-like fold and contains a conserved DEDH motif (D597, E637, D669 and H807) that coordinates two divalent Mg²⁺ ions to hydrolyze the phosphodiester bond in the target RNA^55,57^ (Fig. 4b). The N-terminal domain facilitates duplex unwinding during RISC assembly, thereby assisting in guiding strand selection and the structural rearrangements necessary for catalytic activation^55^. Upon formation of a guide–target duplex with near-perfect complementarity, the target RNA is positioned so that its scissile phosphate aligns within the PIWI catalytic groove, enabling site-specific endonucleolytic cleavage^55,57^. This domain-integrated mechanism underlies AGO2’s specificity and catalytic efficiency in post-transcriptional gene silencing (Fig. 4a, b).

### Diseases associated with AGO mutations

Mutations in the human AGO2 gene are implicated in several diseases, particularly neurodevelopmental disorders. A notable condition is Lessel–Kreienkamp syndrome (LESKRES), which is also known as AGO2 syndrome, an autosomal dominant neurodevelopmental disorder characterized by intellectual disability, delayed motor development, speech and language impairments, and seizures. LESKRES is associated with heterozygous AGO2 mutations that impair RNAi, leading to widespread dysregulation of gene expression during neural development^58,59^.

AGO2 mutations have also been linked to other pathological conditions. AGO2 regulates α-synuclein expression, a protein central to Parkinson’s disease pathogenesis. Reduced AGO2 expression has been observed in the substantia nigra of patients with Parkinson’s disease, suggesting its involvement in disease progression^60^. In addition, AGO2 contributes to cardiac remodeling; in failing hearts, increased nuclear AGO2 localization activates the transcription of genes such as ANKRD1, promoting pathological myocardial remodeling^61^. These findings underscore AGO2’s essential role in maintaining physiological homeostasis and its potential to contribute to disease when dysregulated.

Polymorphisms in AGO genes have also been associated with cancer progression. The rs636832 variant in AGO1 is linked to lymph node infiltration, distant metastasis, advanced clinical stages, recurrence and shorter overall survival in patients with breast cancer. Similarly, the rs2977490 polymorphism in AGO2 is associated with high-grade tumor phenotypes, suggesting a role in tumor aggressiveness. These genetic variations underscore the significance of AGO proteins in both neural development and oncogenesis, warranting further investigation into their functional and pathological implications^62,63^.

### Pathogenic mutations in developmental disorders and cancer

Mutations in human AGO2 have been implicated in neurodevelopmental disorders and cancers^64^. Catalytic residues within the PIWI domain (D597, E637, D669 and H807) are essential for RNA slicing, coordinating Mg²⁺ ions required for phosphodiester bond cleavage (Fig. 4c, d). Pathogenic mutations at these sites, as reported in genomic variant databases such as gnomAD and ClinVar, are extremely rare or absent, probably due to embryonic lethality. By contrast, noncatalytic variants in adjacent regions have been associated with neurodevelopmental disorders. Several mutations identified in LESKRES are structurally significant based on their proximity to the catalytic pocket and RNA-binding interfaces (Fig. 4c). Variants such as L192P, G201C, G201V, H203Q, T357M, M364T, A367P, G573S, C751Y and S760R have been reported in patients with LESKRES, who present with intellectual disability, speech and motor delays, seizures and autism-like behaviors^59^. Notably, mutation hotspots have been identified in the L1 and L2 linker regions (for example, H203Q, T357M, M364T and A367P), which are not traditionally classified as functional domains (Fig. 4e). A cluster of mutations, including T357M, M364T and A367P, is located within an α-helix of the L2 region that connects the MID and PAZ domains (Fig. 4e). Although these residues do not directly interact with other AGO2 domains, the mutations may disrupt local helical stability and misalign the PAZ domain, impairing guide RNA engagement (Fig. 4e). Additional mutations, including H203Q and G201C, are positioned in the L1 region (Fig. 4f). H203 interacts with the phosphate backbone of the target RNA; substitution with glutamine may weaken this interaction. G201, located in a β-strand of L1, may undergo secondary structure disruption when mutated to cysteine, potentially affecting AGO2 folding.

Mutations associated with LESKRES in the MID and PIWI domains (G573S, C751Y, G733R and S760R) also appear to impact interdomain interactions with the L1 and L2 linkers (Fig. 4g, h). Variants such as G733R, C751Y and S760R may introduce steric clashes with the adjacent L2 region, destabilizing the overall structure (Fig. 4g). G573, located in the MID domain, contributes to a β-sheet-like arrangement; its substitution with serine may disrupt this structural element and reduce AGO2 stability (Fig. 4h). These findings highlight the critical role of AGO2’s structural stability, including the linker regions, in preserving its functional integrity.

## Conclusions and prospects

This Review systematically examines the structural impact of disease-associated mutations in RNAi proteins and categorizes them based on their effects on catalytic activity, macromolecular interactions and conformational stability. Mutations in DROSHA, DICER and AGO2, which form the miRNA processing machinery at multiple mechanistic levels, lead to aberrant post-transcriptional regulation and a range of pathologies^18,44,59^.

**Fig. 5:**
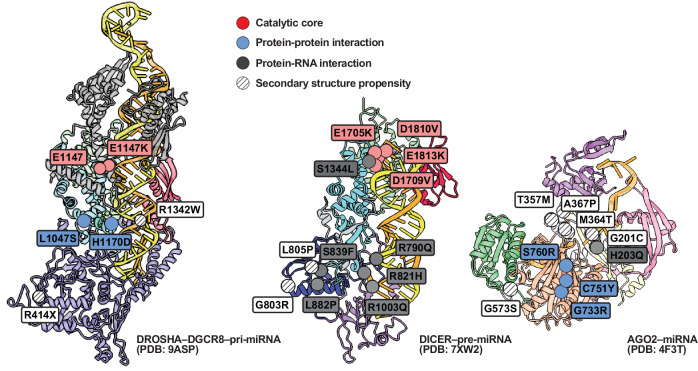
Structural and functional mapping of disease-associated mutations in the human RNAi machinery. Structural localization of pathogenic mutations in DROSHA, DICER and AGO2. Cryo-EM and crystal structures of human DROSHA–DGCR8–pri-miRNA (PDB: 9ASP, left), DICER–pre-miRNA (PDB: 7XW2, center) and AGO2–miRNA (PDB: 4F3T, right) complexes are shown. RNA is rendered in yellow, and protein domains are colored according to the scheme in Fig. 1. Mutation sites are highlighted as spheres and labeled with residue IDs. Mutations are classified by functional consequence and color-coded as follows: catalytic core (red), protein–protein interaction interface (blue), protein–RNA interaction interface (gray) and secondary structure propensity (white stripes). Key residues are annotated with black-bordered labels, and duplicated wild-type catalytic residues are indicated where relevant. X denotes a stop codon.

These mutations can be broadly classified: catalytic inactivation, disruption of protein–protein or protein–RNA interactions, and perturbation of secondary structural elements. Catalytic mutations within the RNase III domains of DROSHA (D1219G and E1147K) and DICER (D1810V, E1705K, E1813K and D1709V) directly impair RNA cleavage by altering residues essential for metal ion coordination. By contrast, catalytic mutations are absent in AGO2, probably reflecting evolutionary constraints on its essential slicing activity (Fig. 5 and Table 1).

**Table 1:** Summary of disease-associated mutations in the human RNAi machinery.

Protein	Mutations	Associated disease	Structural/functional mechanism
DROSHA	E1147K D1219G	Wilms tumor; neurodevelopmental disorders	Catalytic inactivation: disrupts Mg²⁺ coordination in RIIIDb, preventing cleavage
	R414X	Wilms tumor	Truncation: premature stop codon leads to loss of catalytic domains
	L1047S, H1170D	MDS	Destabilization: disrupts hydrophobic packing and π-stacking, impairing folding
DICER	E1705K, D1709V, D1810V, E1813K	DICER1 syndrome; PPB	RIIIDb defect: metal ion coordination failure; leads to 5p-strand loss (strand bias)
	G803R, L805P, S839F	DTPS	Platform disruption: impairs 5′-phosphate binding and substrate alignment
	S1344L	Rhabdomyosarcoma-like tumors	Interface disruption: distorts RIIIDa/b communication
AGO2	T357M, M364T, A367P	LESKRES	Linker instability: destabilizes L2 linker helix, misaligning the PAZ domain
	G201C, H203Q	LESKRES	L1 loop disruption: weakens interaction with target RNA phosphate backbone
	G573S	LESKRES	MID domain instability: disrupts β-sheet structure essential for structural integrity

This table lists recurrent pathogenic mutations in DROSHA, DICER and AGO2 identified in various human disorders, including Wilms tumor, DICER1 syndrome and LESKRES. The corresponding structural and functional consequences—such as catalytic inactivation, truncation or domain destabilization—are summarized for each mutation group based on current structural evidence.

**Fig. 6:**
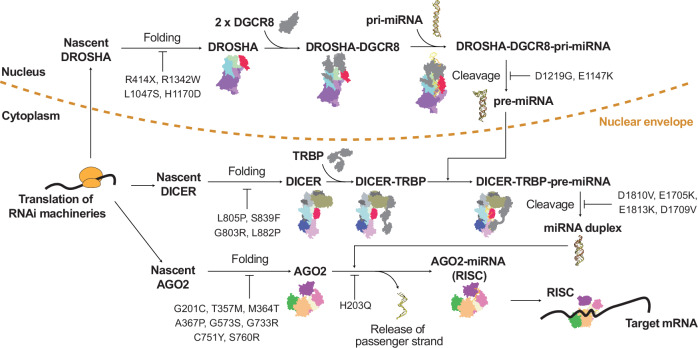
Schematic overview of the intracellular localization, maturation and functional impact of mutations in RNAi pathway components. Each RNAi protein (DROSHA, DICER and AGO2) is translated in the cytoplasm and undergoes correct folding before forming functionally competent complexes. Disease-associated mutations are shown at their respective processing stages, highlighting their effects on protein maturation, complex formation, substrate cleavage and RNAi. Representative mutations are labeled below each protein. Drosha–DGCR8 processes pri-miRNA in the nucleus; Dicer–TRBP cleaves pre-miRNA in the cytoplasm; AGO2 incorporates the miRNA guide strand and mediates RISC assembly and target mRNA repression. The coloring of each domain follows the scheme used in previous figures.

Mutations at protein–protein interfaces, such as L1047S and H1170D in DROSHA, G803R and L882P in DICER, and G733R, C751Y and S760R in AGO2, destabilize interdomain interactions or disrupt cofactor assembly, weakening the structural framework required for RNP complex formation and guide RNA loading. Protein–RNA interaction surfaces are also frequent targets of pathogenic mutations. Although no such mutations have been identified in DROSHA, several occur in DICER (S1344L, R790Q, R821H and R1003Q) and AGO2 (H203Q), probably affecting substrate positioning, cleavage-site selection or guide–target duplex stabilization (Fig. 6).

As the structural resolution of RNAi components advances, structure-guided precision medicine is becoming increasingly feasible. Detailed mapping of mutation sites enables the classification of dysfunction, distinguishing between catalytic loss and structural destabilization. For mutations that destabilize domain interfaces (for example, in the DROSHA belt or DICER platform), small-molecule stabilizers could be designed to reinforce these hydrophobic pockets and restore proper folding^41,51^. Conversely, for mutations that alter RNA-binding affinity without abolishing catalysis, RNA mimetics or antisense oligonucleotides could be engineered to guide the mutant machinery toward its correct substrates^52^. Furthermore, recent explorations into allosteric modulators that can unlock the ‘closed’ conformation of mutant DICER variants offer a promising avenue for restoring miRNA biogenesis in DICER1 syndrome^16^. The integration of cryo-EM data with high-throughput screening will be pivotal in translating these structural insights into viable clinical therapies.
